# Towards visible light driven photoelectrocatalysis for water treatment: Application of a FTO/BiVO_4_/Ag_2_S heterojunction anode for the removal of emerging pharmaceutical pollutants

**DOI:** 10.1038/s41598-020-62425-w

**Published:** 2020-03-24

**Authors:** Benjamin O. Orimolade, Omotayo A. Arotiba

**Affiliations:** 1 Department of Chemical Sciences, University of Johannesburg, Johannesburg, South Africa; 2 Centre for Nanomaterials Science Research, University of Johannesburg, Johannesburg, South Africa

**Keywords:** Catalysis, Electrochemistry, Environmental chemistry, Materials chemistry, Photochemistry, Physical chemistry, Surface chemistry

## Abstract

Pharmaceuticals have been classified as emerging water pollutants which are recalcitrant in nature. In the quest to find a suitable technique in removing them from contaminated water, photoelectrocatalytic oxidation method has attracted much attention in recent years. This report examined the feasibility of degrading ciprofloxacin and sulfamethoxazole through photoelectrocatalytic oxidation using FTO-BiVO_4_/Ag_2_S with p-n heterojunction as anode. BiVO_4_/Ag_2_S was prepared through electrodeposition and successive ionic layer adsorption/reaction on FTO glass. Structural and morphological studies using XRD, SEM, EDS and diffusive reflectance UV-Vis confirmed the successful construction of p-n heterojunction of BiVO_4_/Ag_2_S. Electrochemical techniques were used to investigate enhanced charge separation in the binary electrode. The FTO-BiVO_4_/Ag_2_S electrode exhibited the highest photocurrent response (1.194 mA/cm^−2^) and longest electron lifetime (0.40 ms) than both pristine BiVO_4_ and Ag_2_S electrodes which confirmed the reduction in recombination of charge carriers in the electrode. Upon application of the prepared FTO-BiVO_4_/Ag_2_S in photoelectrocatalytic removal of ciprofloxacin and sulfamethoxazole, percentage removal of 80% and 86% were achieved respectively with a low bias potential of 1.2 V (vs Ag/AgCl) within 120 min. The electrode possesses good stability and reusability. The results obtained revealed BiVO_4_/Ag_2_S as a suitable photoanode for removing recalcitrant pharmaceutical molecules in water.

## Introduction

Water pollution is a global challenge with a lot of negative environmental and health implications. Pollution has the likelihood of increasing owing to increase in industrial activities, improper discharge of household effluents, inefficient wastewater treatment of polluted water and so on. Recalcitrant organic compounds such as pharmaceuticals constitute a major class of emerging water pollutants. Pharmaceuticals, particularly antibiotics have been reportedly found in wastewater and groundwater^1^. When such antibiotics are present in water, they pose serious danger to aquatic organisms and continuous consumption of such water by human can also result in chronic health issues such as the development of strains of bacteria that are resistant to antibiotics^2^. Over the past decades much attention has been directed to developing water treatment methods that are efficient and environmentally friendly since methods based on conventional wastewater treatments often lead to secondary pollutions and incomplete removal of target pollutants in water^3,4^. A recent approach for removing these recalcitrant organics from wastewater is photoelectrocatalytic (PEC) oxidation using suitable semiconducting materials as photoanodes^5,6^.

Photoelectrocatalytic oxidation is an environmentally friendly approach that uses both photon and electric energy to generate powerful oxidants (such as hydroxyl radical) that attack and destroy organic molecules that are present in aqueous solution. When this technique is used to treat water contaminated with recalcitrant organic molecules such as pharmaceuticals, total mineralization to water and carbon dioxide can be achieve over a period of time or the molecules can be broken down to non-toxic organic molecules within a short period of time^7^. Another interesting advantage of this approach is that the use of bias potential results in significant reduction in the challenge of rapid and spontaneous recombination of charge carriers that is peculiar to photocatalysis^8^. Titanium dioxide (TiO_2_) and zinc oxide (ZnO) remained the most applied semiconducting photocatalyst as anodic material for photoelectrocatalytic degradation of organics^9–11^. Owing to the wide band gaps of TiO_2_ (3.2 eV) and ZnO (3.5 eV), they perform best with the application of UV light but the UV region accounts for less than 5% of the solar spectrum^12^. Therefore, other sources of UV light which are expensive are often needed when using TiO_2_ and ZnO. In order to cut down the cost associated with the operation of photoelectrocatalytic degradation process, solar light has been considered as a source of photon energy but this requires that the anodic material be made up of visible light active photocatalyst. In this line, semiconductor photocatalysts such as WO_3_^13^, CuI^14^, Ag_3_VO_4_^15^, Cu_2_O^16^, BiVO_4_^17,18^, Fe_2_O_3_^19^, CuS^20^, Ag_3_PO_4_^21^, WS_2_^22^ and C_3_N_4_^23,24^ have been studied for PEC processes.

In the large pool of visible light active semiconductors, monoclinic sheelite bismuth vanadate (m-BiVO_4_) has proven to be a choice material for PEC applications. As an n-type semiconductor, with narrow band gap (2.4 eV), BiVO_4_ possesses impressive photocatalytic activity under the application of solar light, it is is non-toxic and has good stability. m-BiVO_4_ has been employed as anodic material for the degradation of pollutants in wastewater^6^. It has also found application in PEC water splitting for hydrogen evolution^25^. Unfortunately, the use of unmodified BiVO_4_ is faced with the problem of poor transport of charge carriers as well as relatively fast recombination of photo-excited charge carriers. Over the years, researchers have employed several techniques to counter this problem which include doping with metallic and/or non-metallic impurities, preparation nanosized BiVO_4_ with well-defined morphology, loading of catalyst and formation of heterojunctions with other semiconductors^26,27^. Among these approaches, the formation of heterojunction with other semiconductors have proven to be the most effective.

Basically, heterojunction is formed when two semiconductors of unequal band gap combined in such a way that it results in band alignment^28^. It has been observed that the formation of heterojunctions between p-type and n-type semiconductor can improve PEC activity through improved light harvesting, effective separation of photogenerated electron-hole pairs and thus increased the lifespan of the charge carriers. For instance, Soltani *et al*.^29^, prepared BiFeO_3_/BiVO_4_ with p-n heterojunction through facile ultrasonic/hydrothermal route and they observed improved charge separation in the composite as shown in the current density of 0.23 mA/cm^−2^ achieved on BiFeO_3_/BiVO_4_ which was three times higher than that of pristine BiVO_4_. Additionally, higher percentage degradation of tetracycline was reported with the application of the prepared BiFeO_4_/BiVO_4_ p-n heterostructure. Likewise, similar observations have been demonstrated in other BiVO4 based p-n heterostructures such as Cu_2_O/BiVO_4_^30^, BiVO_4_/MnO_2_^31^, BiVO_4_/CeVO_4_^32^, WO_3_/BiVO_4_^33^, CdS/BiVO_4_^34^, Fe_2_O_3_/BiVO_4_^35^, BiVO_4_/ZnO^36^, BiVO_4_/NiO^37^, β-AgVO_3_/BiVO_4_^38^ and BiVO_4_/Ag_3_PO_4_^39^.

The selection of an appropriate p-type semiconductor is a critical step to achieve p-n heterojunction of BiVO_4_ with improved performance. Recently, attention has been given to silver sulfide (Ag_2_S) as a suitable semiconductor to form p-n heterojunction with BiVO_4_. As a chalcogenide based p-type semiconductor, Ag_2_S has good optical properties and photocatalytic activity owing to its small band gap (between 0.9–1.1 eV)^40^. BiVO_4_/Ag_2_S p-n heterostructure prepared through hydrothermal routes have shown enhanced photocatalytic performance for the degradation of dyes and pharmaceuticals^41^. Guan *et al*.^42^, have also demonstrated the photoelectrochemical performance of BiVO_4_/Ag_2_S in water splitting and achieved a high photocurrent density of 1.91 mA/cm^2^. To the best of our knowledge, the performance of BiVO_4_/Ag_2_S in photoelectrochemical oxidation of pharmaceuticals in aqueous solution have not been reported.

Herein, we report for the first time the photoelectrocatalytic degradation of pharmaceuticals in water using a p-n heterostructure of BiVO_4_/Ag_2_S prepared on FTO glass as anode. The BiVO_4_/Ag_2_S photoanode with improved PEC performance was prepared on FTO glass using two-step electrodeposition and successive ionic layer adsorption/reaction (SILAR) methods. The optical property of the electrode was studied using UV diffusive reflectance spectroscopy (UV-DRS) while structural and morphological studies were carried out with XRD, SEM and EDS. Chronoamperometry and linear sweep voltammetry were used to confirm improved photocurrent response of the material. Electrochemical impedance spectroscopy and Mott Schottky plot were also used to establish the formation of heterojunction between the two semiconductors. Ciprofloxacin and sulfamethoxazole were selected as pollutant of interest for the photelectrocatalytic degradation experiments.

## Experimental

### Materials and reagents

All the chemicals used were purchased from Sigma Aldrich (South Africa. These include bismuth nitrate pentahydrate (Bi(NO_3_)_3_._5_H_2_O), potassium iodide, vanadylacetylacetonate, silver nitrate, sodium sulfide, sodium hydroxide pellets, p-benzoquinone, sodium sulfate, potassium hexacyanoferrate (II), potassium hexacyanoferrate (III), ciprofloxacin and sulfamethoxazole.

### Preparation of BiVO_4_/Ag_2_S photoanodes

The binary photoanode with p-n heterojunction was fabricated through a two-step electrodeposition and Successive Ion Layer Adsorption/Reaction methods. First, BiVO_4_ were electrodeposited on a FTO glass (5 cm × 1.3 cm × 0.22 cm, surface resistivity of ~7 Ω/sq) using a modified previously documented electrodeposition technique^34,43^. Summarily, from a well sonicated precursor solution containing 0.49 g Bi(NO_3_)_3_·5H_2_O, 1.66 g KI in 25 mL and 0.23 M p-benzoquinone maintained at pH 4.3, films of BiOI were first potentiostatically electrodeposited onto a clean FTO glass at −0.13 V for 720 s. FTO glass, platinum wire and Ag/AgCl (3.0 M KCl) electrode were employed as the working electrode, counter electrode and reference electrode respectively. After rinsing the obtained BiOI electrode with water several times and drying at room temperature, 100 μL of 0.20 M vanadylacetylacetonate (dissolved in DMSO) was drop-cast evenly onto the BiOI electrode. The electrode was subsequently placed in a furnace at 420 °C for 1 h. Finally, excess V_2_O_5_ was washed off from the electrode by soaking it in 1.0 M NaOH solution for 40 min. The resulting BiVO_4_/FTO electrode was thoroughly washed with deionized water and dried at room temperature. In order to obtain BiVO_4_/Ag_2_S electrode, the prepared FTO/BiVO_4_ was dipped in a 0.3 M AgNO_3_ solution for 10 s and followed by immersion in 0.3 M Na_2_S for 10 s. The cycle was repeated ten times and the obtained electrode was rinsed with deionized water and air dried at room temperature for 24 h.

### Structural and morphology characterisation of the prepared electrodes

X-ray diffractometer (Rigaku Ultima IV, Japan) using Cu Kα radiation (k = 0.15406) with K-beta filter at 30 mA and 40 kV was used to identify the phase, degree of crystallinity and purity of the prepared semiconductor photoanodes. TESCAN Vega 3(Czech Republic) scanning electron microscope was employed to determine surface morphology of the material. Energy-dispersive spectrometer (EDS) attached to the SEM instrument was used to confirm the presence of expected elements in the prepared materials in the appropriate ratio. The light absorption properties of the materials were analyzed using UV/Visible-Diffuse Reflectance Spectroscopy. A similar characterisation methods has been previously reported^13^.

### Electrochemical and photoelectrochemical experiments

The electrochemical and photoelectrochemical experimental protocols are similar to that described in our previous reports^8,43^. Photocurrent measurements, linear sweep voltammetry (LSV) and electrochemical impedance spectroscopy (EIS) were performed on an Autolab PGSTAT204 (Netherlands) potentiostat/galvanostat. The working electrodes were the prepared BiVO_4_, Ag_2_S and BiVO_4_/Ag_2_S electrodes. Platinum sheet with equal dimension as the FTO glass was employed as counter electrode while the reference electrode was Ag/AgCl (3.0 M KCl). Chronoamperometry and LSV were carried out in a 0.1 M Na_2_SO_4_ solution. EIS was done in a 5 mM solution of [Fe(CN)_6_]^3−/4−^ (prepared in a 0.1 M KCl solution). Data for Mott Schottky plots were obtained under dark condition at room temperature. For photoelectrochemical experiments, a solar simulator equipped with a 100 W xenon lamp was used as the light source. The prepared electrode was fixed vertically facing the incident light of the simulator and the distance between the photoelectrochemical cell and the light source was 10 cm and the glass were illuminated from the rear. The experiments were performed in a 70 mL capacity reactor made of quartz glass. For the degradation experiments, the working solution was 50 mL solution of 0.1 M Na_2_SO_4_ (supporting electrolyte) and 10 mgL^−1^ of the pharmaceuticals. Aliquots of the solution taken from the reactor at predefined time intervals using a disposable syringe were analyzed using UV–Visible spectrophotometer to obtain the concentration decay pattern. The total organic carbon was also measured using TOC analyser (Teledyne Tekmar TOC fusion). The applied bias potential was optimized by performing the PEC experiments at different bias potential.

## Results and discussion

### Structural and morphology characterization of the electrodes

**Figure 1:**
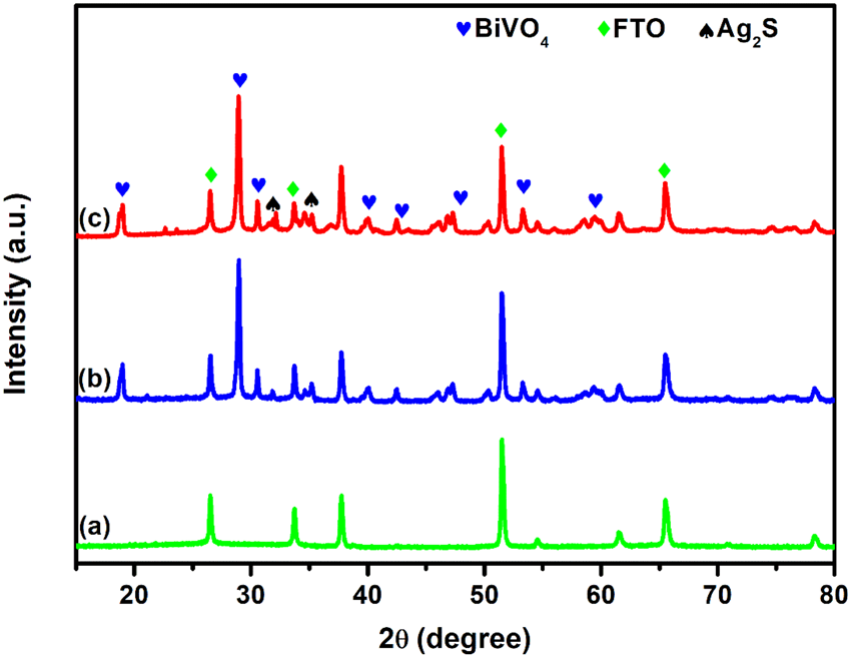
XRD patterns of (**a**) FTO, (**b**) FTO/BIVO_4_ and FTO/BiVO_4_/Ag_2_S.

The X-ray diffractograms of the prepared photoanodes are presented in Fig. 1. All the peaks correspond to those of monoclinic scheelite BiVO_4_ (JCPDS no. 75–1866) in the XRD pattern of the BiVO_4_. The main peaks at 18.8°, 28.73°, 30.64°, 34.01°, 35.06°, 39.95° and 42.35°can be indexed as (110, 011), (121), (040), (200), (002), (211) and (150) crystal planes respectively^44^. In the XRD pattern of BiVO_4_/Ag_2_S, the diffraction peaks of Ag_2_S are not pronounced and well visible which could suggest that the particles are well dispersed on the surface of the BiVO_4_ or probably due to relatively lower content and low crystallinity of Ag_2_S loading^45^. Nonetheless, the presence of Ag_2_S in the sample is evident in the peaks at 32° and 35° which appeared to be superimposed on those of BiVO_4_ and therefore changing their intensities^46^. Expectedly, all the characteristic peaks BiVO_4_ were still observed in the XRD pattern of the BiVO_4_/Ag_2_S electrode. In order to further confirm the presence of Ag_2_S on the binary electrode, other morphological studies were carried out.

**Figure 2:**
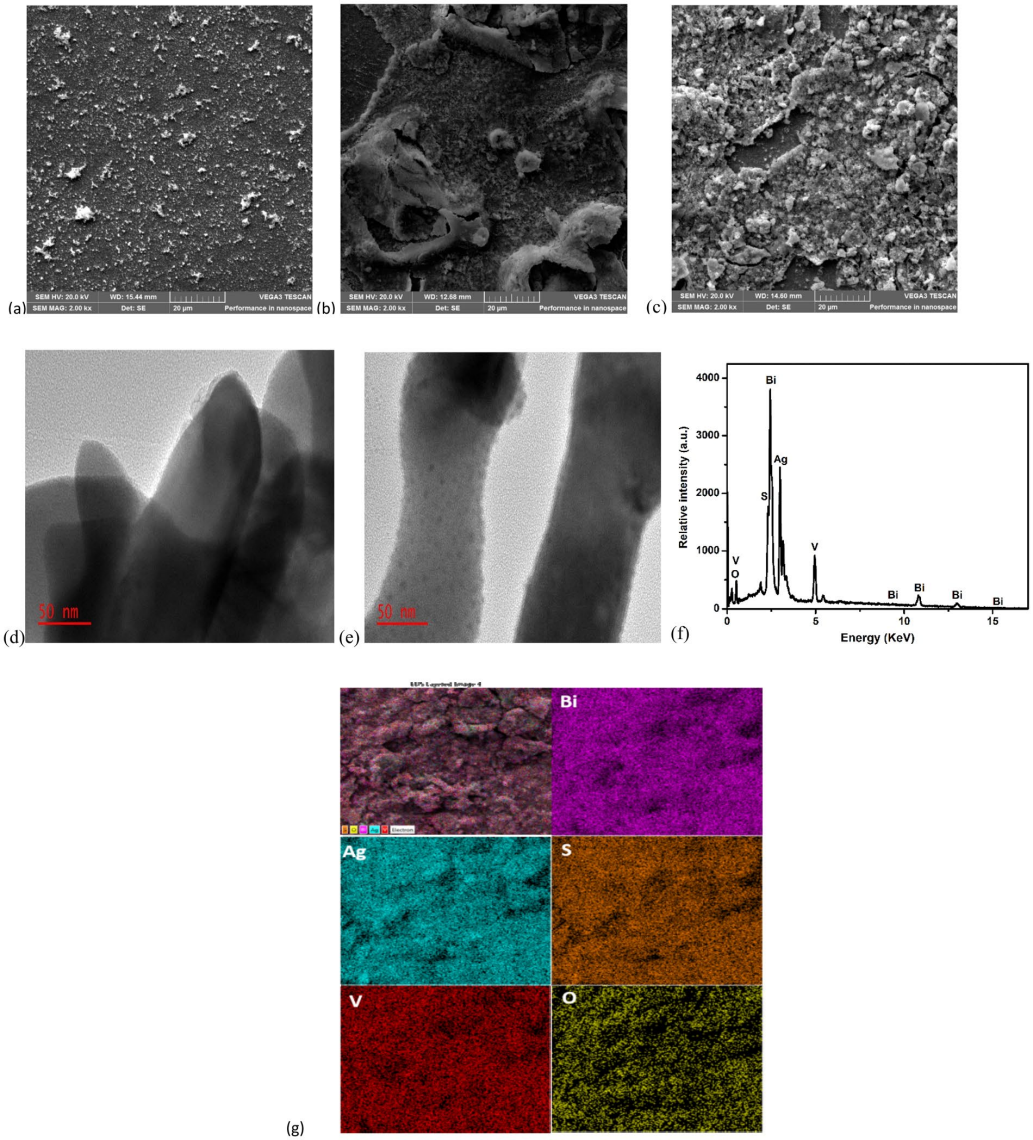
SEM images of (**a**) FTO-Ag_2_S; (**b**) FTO-BiVO_4_; (**c**) FTO-BiVO_4_/Ag_2_S; HR-TEM images of (**d**) BiVO4; (**e**) BiVO4/Ag2S; (**f**) EDS spectrum of FTO-BiVO_4_/Ag_2_S and (**g**) EDS elemental mapping of FTO-BiVO_4_/Ag_2_S electrode.

The surface morphology of the photoanodes prepared on FTO glass are shown in Fig. 2(a–c). The prepared Ag_2_S appears as fine particles dispersed on the FTO glass (2a) while the particles of BiVO_4_ are agglomerated on the FTO glass forming film like structure (2b). The incorporation of Ag_2_S particles onto the BiVO_4_ on FTO glass resulted in agglomerated globules with openings which could serve as active sites for capturing of target analytes as shown the SEM image of FTO-BiVO_4_/Ag_2_S (Fig. 2c) and this further established that Ag_2_S were successfully coupled with the electrodeposited BiVO_4_. HR-TEM was further used to evaluate the nanostructure and heterostructure interface property between BiVO_4_ and Ag_2_S in the BiVO_4_/Ag_2_S composite and the from the results it can be seen that BiVO_4_ materials appeared as nanorods of different sizes (Fig. 2d) while Ag_2_S were nanoparticles which were well dispersed on the surface of BiVO_4_ nanorods (Fig. 2e) suggesting the successful formation of appropriate heterostructure interface. As shown in Fig. 2f, the EDS spectrum revealed that only Bi, V, O, Ag and S were present in the composite electrode suggesting that the material is reasonably pure since no unwanted element was observed in the spectrum. Additionally, the percentage composition of each element obtained from the result also agreed with theoretical calculation of the elemental composition of BiVO_4_/Ag_2_S composite. It is also interesting to note that distribution of the elements on the electrode surface is uniform as revealed in the EDS mapping (Fig. 2g) and this further confirmed the evenly spread of Ag_2_S particles on the electrodeposited BiVO_4_.

### Optical properties of the photoanodes

**Figure 3:**
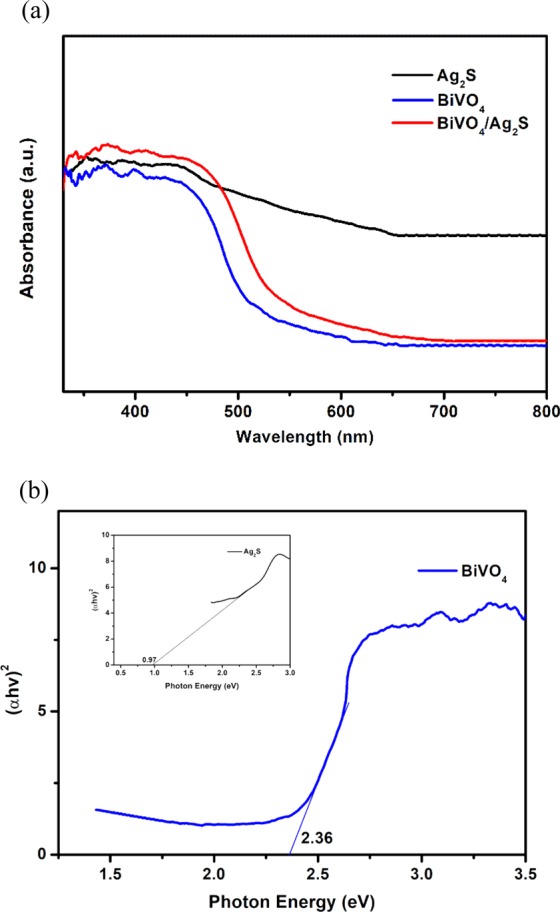
(**a**) UV-Visible diffuse reflectance spectra and (**b**) the energy band gap edges of Ag_2_S, BiVO_4_ and BiVO_4_/Ag_2_S electrodes.

The optical properties of the prepared Ag_2_S, BiVO_4_ and BiVO_4_/Ag_2_S electrodes were studied using UV-Visible diffuse reflectance spectroscopy and the results are shown in Fig. 3a. All the electrodes absorb photons in the visible light region and the absorption edges can be traced to 540 nm and 620 nm for BiVO_4_ and BiVO_4_/Ag_2_S respectively while the absorption edge of Ag_2_S tends towards the near infrared. The shift of the absorption edge of BiVO_4_/Ag_2_S and increase in absorption can rightly be attributed to the enhancement of BiVO_4_ optical ability through the addition of Ag_2_S. In order determine the band gap energy of the two semiconductors, the data obtained from the UV-DRS analyses were fit into Tauc equation which established that the band gap energy of a semiconductor can be determine using Eq. (1).

1 $$\alpha hv=A{(vh-{E}_{g})}^{n/2}$$

where α, h, A, E_g_ and v are the absorption coefficient, Planck’s constant, constant, band gap energy and incident light frequency respectively; ‘n’ is a constant that depends solely on the optical transition characteristics of the semiconductors under consideration. For direct transition semiconductors the value of ‘n’ is 1^47^. Since both BiVO_4_ and Ag_2_S are direct semiconductors, a plot of (αhv)^2^ against hv was made from which the value of E_g_ for BiVO_4_ and Ag_2_S were estimated to be 2.36 eV and 0.97 eV respectively (Fig. 3b). The values for the band gap energies obtained for both BiVO_4_ and Ag_2_S are in agreement with previously reported values for the semiconductors^48,49^. These results further confirm the successful preparation of visible light active semiconductor photocatalysts. The improved photoabsorption in BiVO_4_/Ag_2_S suggested enhanced charge separation through band alignment when BiVO_4_ and Ag_2_S combined together and this was further established through series of photoelectrochemical experiments.

### Electrochemical and photoelectrochemical analysis

**Figure 4:**
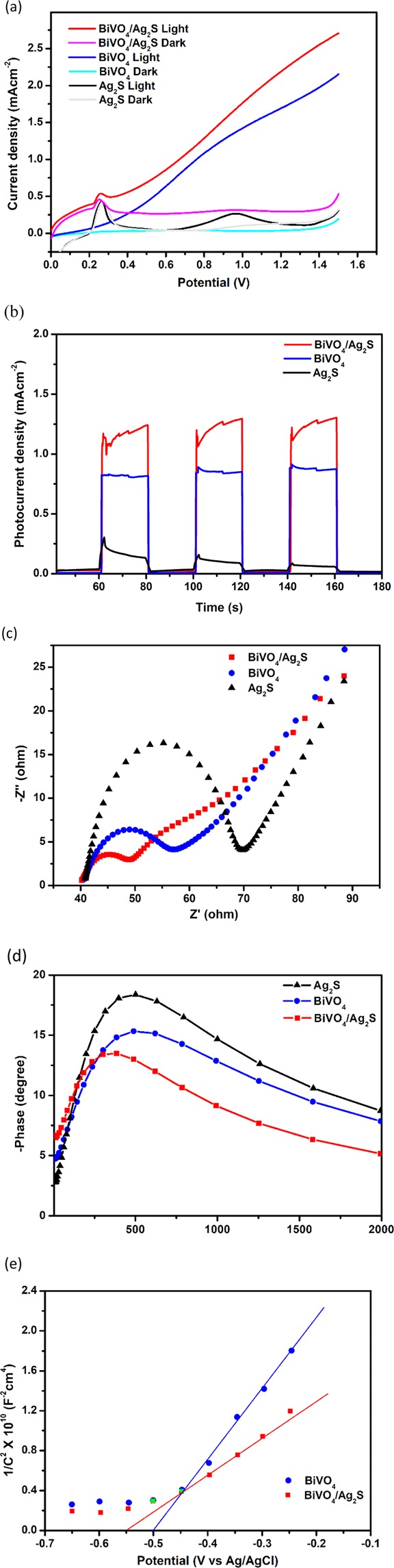
(**a**) Linear sweep voltammogram at 20 mVs^−1^; (**b**) photocurrent response in 0.1 M Na_2_SO_4_; (**c**) Nyquist EIS plot; (**d**) Bode plot and (**e**) Mott Shottky plot for Ag_2_S, BiVO_4_ and BiVO_4_/Ag_2_S photoanodes in 5 mM [Fe(CN)_6_]^3−/4−^ in 0.1 M KCl (pH 7).

Linear sweep voltammetry (LSV) of the photoanodes were carried out in a solution of 0.1 M Na_2_SO_4_ (pH 7) at a scan rate of 20 mVs^−1^. The linear voltammograms (Fig. 4a) were recorded in both the presence and absence of visible light illumination. All the electrodes showed higher current responses with illumination than without illumination which could be attributed to that fact that when the materials were irradiated, there is instantaneous excitation of electrons from the valence band to the conduction band and this enhanced better conductivity. Ag_2_S shows improved responses at 0.28 V and 0.85 V while BiVO_4_ show a continuous increase in photocurrent response with increase in potential. Interestingly, though the binary electrode of BiVO_4_/Ag_2_S showed the characteristics features of both the voltammogram obtained with Ag_2_S and BiVO_4_ (Fig. 4a) its overall increase in photocurrent response with increase in potential was higher than both the pristine electrode suggesting a good improvement in charge separation resulting in higher light responsiveness through the construction heterointerface. The anodic peak at ca 250 mV (Fig. 4a) is due to the oxidation of silver. This peak occurs only in Ag containing electrodes and thus a further confirmation of the presence of Ag_2_S in the heterojunction electrode BiVO_4_/Ag_2_S.

It has been also been established that there is a linear correlation between the transient photocurrent of a semiconducting material and the charge separation process taking place within the material^13^. Therefore, with applied external potential of +0.8 V (selected based on the LSV performance of the electrodes), the transient photocurrent responses of the electrodes were recorded using chronoamperometry method (Fig. 4b). As expected, the highest photocurrent (1.194 mA/cm^−2^) was attained with BiVO_4_/Ag_2_S electrode which was significantly higher than that of pristine BiVO_4_ (0.802 mA/cm^−2^) and almost ten times greater than that of Ag_2_S (0.165 mA/cm^−2^). Therefore, it was clear that the construction of p-n heterojunction between BiVO_4_ and Ag_2_S promotes charge transfer between the interfaces of the two semiconductors which greatly inhibit the rapid recombination of photogenerated electron – hole pairs in the BiVO_4_/Ag_2_S electrode.

The results obtained with electrochemical impedance spectroscopy further corroborate the improved performance of BiVO_4_/Ag_2_S heterojunction through synergistic effect of both BiVO_4_ and Ag_2_S. The experiments were performed in an electrolytic solution of 5 mM [Fe(CN)_6_]^3−/4−^ in 0.1 M KCl (pH 7) with external application of +0.2 V. The obtained Nyquist plots for the fabricated photoanodes are displayed in Fig. 4c. For all the electrodes single characteristic semicircles were obtained in the EIS spectra which signifies the charge transfer process happening at the solution-electrode interface. The size of the semi-circular arc in the spectra is a function of the charge-transfer resistance (R_ct_) at the interface of the heterojunction and studies have shown that the smaller the arc radius the better the charge transfer efficiency^50,51^. Accordingly, the lowest R_ct_ was obtained from the Nyquist plot of BiVO_4_/Ag_2_S. This further affirmed that the formation of heterojunction between BiVO_4_ and Ag_2_S resulted in better charge mobility and lowered rate of instantaneous recombination of photogenerated electron-hole pairs. Furthermore, the impedance data were analyzed with a bode phase angle plot (Fig. 4d) d to determine the electrons lifetime and charge transfer resistance in the BiVO_4_/Ag_2_S electrode. As shown in Fig. 4d, the maximum phase angle of the heterojunction electrode shifts to lowest frequency as compared to both Ag_2_S and BiVO_4_. This confirms the rapid electron transport process happening in the heterojunction. The life time of electrons is related to the frequency as given in Eq. (2)^52^.

2 $${\tau }_{\varepsilon }=1/2\pi {f}_{max}$$

where f_max_ is the frequency at the maximum phase angle the bode plot. Using Eq. (2), the electron lifetime of BiVO_4_/Ag_2_S was calculated to be 0.40 ms which was longer than those of BiVO_4_ (0.32 ms) and Ag_2_S (0.31 ms). This value of life time calculated further confirms that the fabrication of the heterojunction helped in minimizing rapid recombination of electron – hole pairs in the two semiconductors through a fast charge transfer process^53^.

The flat band potential (E_FB_) and charge carrier density (N_D_) of a semiconductor can also be used as a measure of improved charge separation in semiconductor – semiconductor heterojunction interfaces = These values can be obtained from potential scan measurements and fitting of data to obtain Mott Schottky plot. Mott Schottky equation is given in Eq. 3.

3 $$1/{C}^{2}=2/(e{{\rm{\varepsilon }}{\rm{\varepsilon }}}_{0}{N}_{D})\cdot ({E}_{app}-{E}_{FB}-kT/e)$$

C, e, ɛ, ɛ_0_, E_app_, T, N_D_, E_FB_ and k represent the capacitance at the semiconductor/electrolyte interface (Fcm^−2^), elementary charge (1.60 × 10^−19^ C), dielectric constant (68 for BiVO_4_^54^), permittivity of vacuum, external applied potential, absolute temperature, donor density, flat band potential and Boltzmann constant respectively.

From Eq. (3), a plot of 1/C_2_ against E_app_ was constructed and Donor density (N_D_) was calculated from the slope while the approximately value of flat band potential was extrapolated from the intercept (Fig. 4e). As an n-type semiconductor, the MS plot of BiVO_4_ gave a positive slope value. A negative shift in the flat band potential from −0.512 V in BiVO_4_ to −0.548 V in BiVO_4_/Ag_2_S was also observed and this suggested that the rate of rapid recombination of charge carriers in the constructed BiVO_4_/Ag_2_S heterojunction was greatly reduced. This observation was further justified the carrier density of BiVO_4_/Ag_2_S (3.87 × 10^22^ cm^−3^) which was significantly larger than that of pristine BiVO_4_ (8.07 × 10^21^ cm^−3^).

### Photoelectrocatalytic degradation of pollutants

**Figure 5:**
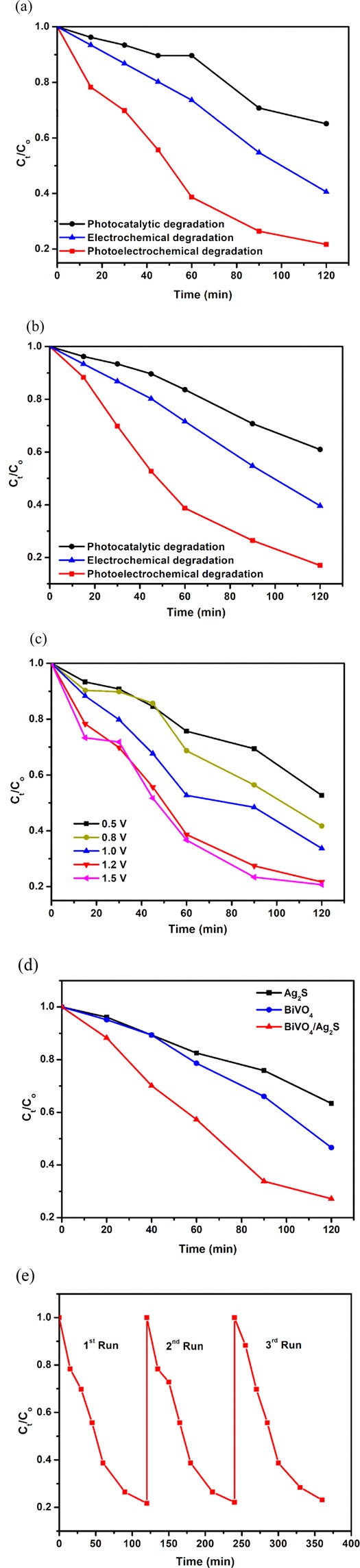
(**a**) Normalised concentration decay versus time plot for photocatalytic, electrocatalytic and photoelectrocatalytic degradation of (**a**) ciprofloxacin and (**b**) sulfamethoxazole using FTO-BiVO_4_/Ag_2_S electrode; (**c**) Effects of potential on degradation of ciprofloxacin; (**d**) Normalised concentration decay versus time plot for PEC degradation of ciprofloxacin on FTO-Ag_2_S, FTO-BiVO_4_, FTO-BiVO_4_/Ag_2_S electrodes; (**e**) Cycle experiments for the degradation of ciprofloxacin on FTO-BiVO_4_/Ag_2_S electrode.

The photoelectrocatalytic degradation of organics on the prepared BiVO_4_/Ag_2_S electrode was evaluated by using ciprofloxacin and sulfamethoxazole as target water contaminants. The degradation was achieved with an applied bias potential of 1.2 V, pH 7 and simulated sunlight was used as light source. The degradation processes of the pharmaceuticals were followed using UV-Visible spectrophotometer and evidence of reduction in the concentrations of ciprofloxacin and sulfamethoxazole was seen by the decrease in the intensity of the peaks at 276 nm and 265 nm for ciprofloxacin and sulfamethoxazole respectively. Within 120 min, a percentage removal of 80% and 86% for ciprofloxacin and sulfamethoxazole was achieved (Fig. 5a,b). The breaking down of ciprofloxacin molecules was further confirmed through the percentage total organic carbon removal (TOC) which was 69%. In the absence of light, the percentage anodic electrochemical degradation of ciprofloxacin and sulfamethoxazole were 59% and 61% respectively while percentage degradation achieved with photocatalysis alone was 35% and 40% respectively. The highest degradation achieved with photoelectrocatalytic degradation showed that the application of bias potential in conjunction with photocatalysis facilitated the breaking down of the organic molecules as the bias potential helps in driving away photoexcited electrons from the surface of the photoanode and thereby reducing the occurrence of recombination of the electron – hole pairs.

The kinetics studies also revealed that the degradation processes of both ciprofloxacin and sulfamethoxazole were fastest with the application of photoelectrocatalytic oxidation (Figs. S1 and S2). It is also interesting to note that the degradation of sulfamethoxazole on the BiVO_4_/Ag_2_S electrode appeared to be more favourable than that of ciprofloxacin as evident with the higher percentage degradation (86%) and supported with the apparent rate constant (0.0147 min^−1^) which was higher than that of ciprofloxacin (80% and 0.0137 min^−1^). This could be attributed partly to the larger molecular mass and more complex structure of ciprofloxacin.

Applied bias potential is a critical parameter that affects photoelectrocatalytic degradation process. In order to evaluate the dependence of percentage removal of organics using the binary electrode on potential, the photoelectrocatalytic degradation was carried out with applied bias potential in the range of 0.2 V–1.5 V (Versus Ag/AgCl). It can be seen that the percentage degradation of ciprofloxacin increased with increase in applied bias potential with a value of about 10% stepwise up till potential of 1.2 V (Fig. 5c). The applied bias potential plays a major role in separation electron – hole pair by driving the photogenerated electrons away from the anode towards the cathode. As shown in the result, the higher the applied potential, the higher the driving force for the electron. Therefore, the degradation increases because there was reduced recombination of photoinduced electron – hole pairs at higher potential. When higher potential of 1.5 V was applied, the difference in the percentage degradation with that obtained with 1.2 V was approximately 1% which was relatively insignificant when compared with the trend. This revealed that beyond the optimal potential, higher applied bias potential could yield insignificant improvement in the percentage degradation which could be due to side reaction of evolved oxygen at higher potential^55^. Based on the result obtained, 1.2 V was selected as the optimal bias potential for the photoelectrocatalytic degradation of pharmaceuticals on the BiVO_4_/Ag_2_S electrode.

The improved charge separation in the binary electrode through the construction of p-n heterojunction was also confirmed by comparing its performance in the photoelectrocatalytic degradation of pharmaceuticals with that of the pristine electrodes of BiVO_4_ and Ag_2_S. As shown in Fig. 5d, BiVO_4_ and Ag_2_S electrodes gave a percentage removal of 63% and 50% respectively which were lower than 80% ciprofloxacin removal achieved on the BiVO_4_/Ag_2_S photoanode. The better performance of the binary electrode suggested that p-n heterojunction constructed facilitated the migration of electrons from the conduction band of Ag_2_S to BiVO_4_ while holes from BiVO_4_ moves to the valence band of Ag_2_S yielding enhanced photogenerated charge carriers separation resulting in better photoelectrocatalytic performance^28^.

One of the advantages of photoelectrocatalytic degradation over convention photocatalysis is the ease of reusability of the material. The BiVO_4_/Ag_2_S electrode also showed impressive stability and reusability as seen from the cycling experiments (Fig. 5e). The results were obtained by using the same BiVO_4_/Ag_2_S three different times. After each cycle, the electrode was purged with deionized water and air dried at room temperature. After the third application the percentage removal of ciprofloxacin was approximately 79% suggesting that there was no remarkable change in the performance of the electrode after using it three times showing that the electrode is relatively stable and can be reused.

### Proposed mechanism of degradation and scavenger studies

The degradation of organic molecules during photoelectrocatalytic processes happens when generated reactive species attack and oxidize the organic molecules. The photogenerated holes, hydroxyl and superoxide radicals usually play the predominant roles in photoelectrocatalytic degradation experiments. Equations 4–9 give the mechanism of formation of these reactive species and their oxidation reactions with the pharmaceutical molecules for total mineralization.

4 $${\rm{hv}}+{{\rm{BiVO}}}_{4}/{{\rm{Ag}}}_{2}{\rm{S}}\to {{\rm{BiVO}}}_{4}/{{\rm{Ag}}}_{2}{\rm{S}}\,{({\rm{h}}}_{{\rm{VB}}}^{+}+{{\rm{e}}}_{{\rm{CB}}}^{-})$$

5 $${{\rm{e}}}_{{\rm{CB}}}^{-}+{{\rm{O}}}_{2}\to {}^{.}{{\rm{O}}}_{2}^{-}$$

6 $${{\rm{h}}}_{{\rm{VB}}}^{+}+{{\rm{H}}}_{2}{\rm{O}}\to {}^{.}{\rm{OH}}+{{\rm{H}}}^{+}$$

7 $$\,{}^{\cdot }{\rm{O}}{\rm{H}}+{\rm{pharmaceutical}}\to {{\rm{CO}}}_{2}+{{\rm{H}}}_{2}{\rm{O}}$$

8 $$\,{}^{\cdot }{\rm{O}}_{2}^{-}+{\rm{pharmaceutical}}\to {{\rm{CO}}}_{2}+{{\rm{H}}}_{2}{\rm{O}}$$

9 $${{\rm{h}}}_{{\rm{VB}}}^{+}+{\rm{pharmaceutical}}\to {{\rm{CO}}}_{2}+{{\rm{H}}}_{2}{\rm{O}}$$

**Figure 6:**
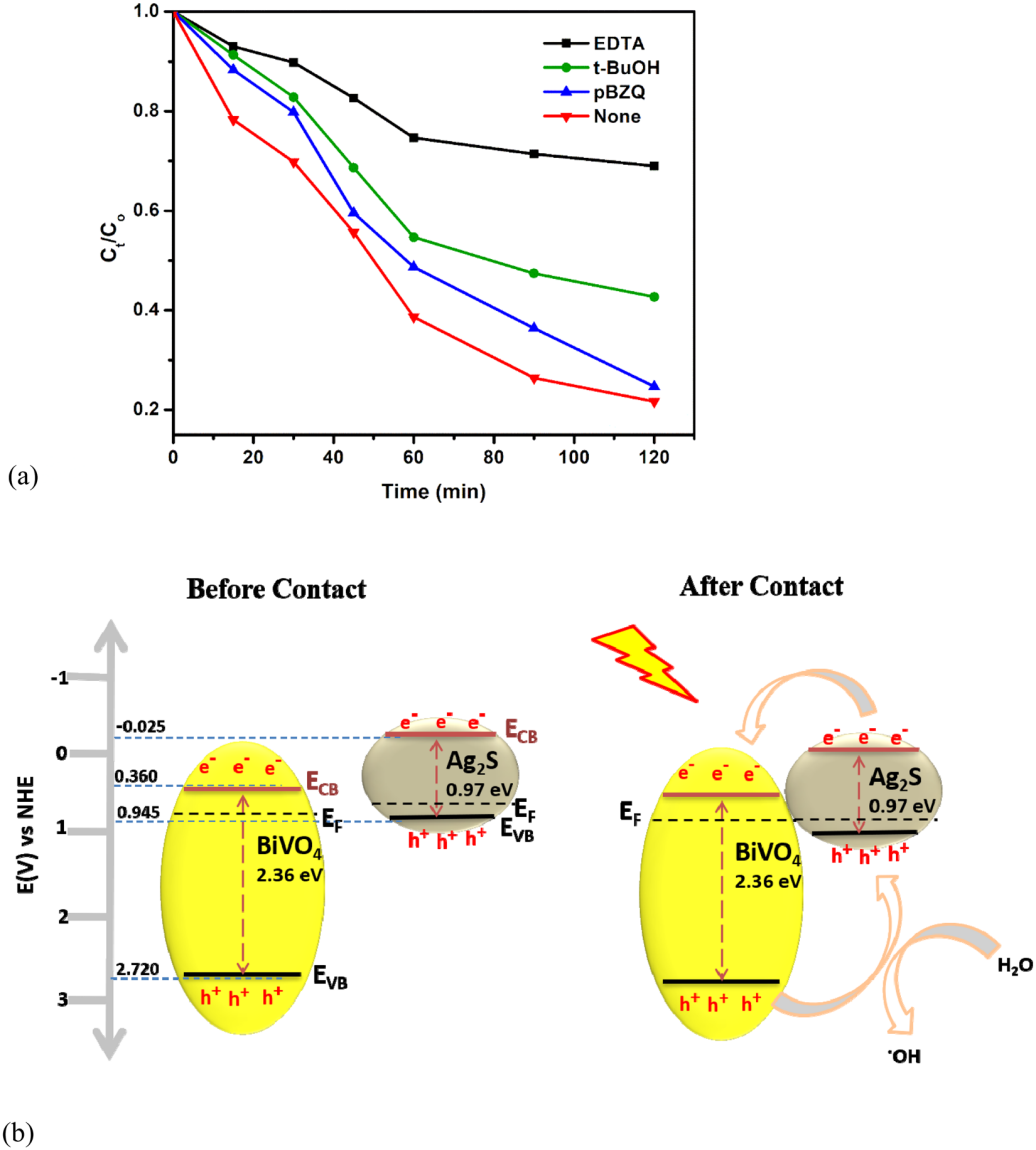
(**a**) Scavenger studies of the photoelectrocatalytic degradation of ciprofloxacin on FTO-BiVO_4_/Ag_2_S; (**b**) Band alignment between BiVO_4_ and Ag_2_S.

The contribution of individual reactive specie in the PEC degradation of the ciprofloxacin molecule was determined by trapping experiments which was conducted by inhibiting the effects of holes, hydroxyl radicals and superoxide radicals through the introductions of ethylenediaminetetraacetate salt (EDTA), t-butanol (t-BuOH) and p-benzoquinone (p-BZQ) respectively^56,57^ in the reaction medium. As seen in Fig. 6a, photogenerated holes play a crucial role in the breaking down of the pharmaceutical molecules since the percentage removal dropped to almost 10% when holes were masked through the addition of EDTA. The effect of hydroxyl radicals on the degradation of the pharmaceutical molecules cannot also be overlooked process because the degradation efficiency dropped to 52% with the addition of t-butanol. The hydroxyl radicals were produced in the reaction system through the oxidation reactions of water molecules by photogenerated holes. Unlike the holes and hydroxyl radicals, superoxide radicals performed a seemingly insignificant role in the oxidation of the pharmaceutical molecules because percentage removal of 76% was still achieved when the superoxide radicals were trapped by the addition of p-BZQ. Literatures have shown that hydroxyl radicals are not produced in detectable amount when BiVO_4_ is illuminated due to rapid recombination because of rapid recombination with photogenerated electrons^48,58^. But in this work, the fact that holes and hydroxyl radicals play predominant roles the breaking down of the pharmaceuticals confirms that better charge separation can be achieved with BiVO_4_ through the formation of heterojunction with Ag_2_S.

The possible mechanism of the spontaneous mobility of photogenerated electron-hole pairs between the interface the two semiconductors is proposed by obtaining the relative band edge potential of the conduction band and valence band of both semiconductors using Eqs. 9 and 10.

10 $${E}_{CB}=X-{E}_{c}-0.5{E}_{g}$$

11 $${E}_{VB}={E}_{g}+{E}_{CB}$$

E_VB_ and E_CB_ stand for the valence and conduction band edge potentials respectively. X represent the electronegativity of the semiconductor usually calculated as the geometric mean of the absolute electronegativities of the constituent atoms in the semiconductor (X = 6.04 for BiVO_4_ and 4.96 for Ag_2_S). E_C_ is the energy of the free electrons on hydrogen scale which is approximately 4.50 eV (vs NHE). Energy band gap (E_g_) has been estimated to be 2.36 eV for BiVO_4_ and 0.97 eV for Ag_2_S respectively using Tauc equation (Fig. 3b). Therefore, the E_CB_ and E_VB_ of BiVO_4_ were calculated to be 0.360 eV and 2.720 eV respectively while for Ag_2_S, the values obtained were −0.025 eV and 0.945 eV for E_CB_ and E_VB_ respectively. The values of E_CB_ and E_VB_ for Ag_2_S are lower than the corresponding values for BiVO_4_ indicating that the formation of type II heterojunction is possible when the two semiconductors aligned. As a p-type semiconductor, the Fermi energy level of Ag_2_S is located slightly above the valence band while that of BiVO_4_, n-type, is slightly below its conduction band. As shown in Fig. 6b, when the two semiconductors are in contact, the Fermi energy level aligned and internal electric field is established in such a way that the holes can be effectively separated into the valence band of Ag_2_S and be available to oxidize directly the pharmaceutical molecules or produce hydroxyl radicals from water molecules while the electrons migrate to the conduction band of BiVO_4_ in opposite direction of the internal electric field resulting in efficient charge separation^28^. The electrons can also react with oxygen molecules to produced superoxide radicals^59^ but the effect superoxide in the degradation process in this study is limited.

## Conclusion

A photoanode of BiVO_4_/Ag_2_S with p-n heterojunction was successful prepared through electrodeposition and successive ionic layer adsorption/reaction method on FTO glass. The construction of p-n heterojunction reduced the common problem of rapid recombination of photogenerated electron-hole pairs. This was confirmed through the photocurrent response of BiVO_4_/Ag_2_S (1.194 mA/cm^−2^) which was higher than both pristine BiVO_4_ (0.802 mA/cm^−2^) and Ag_2_S (0.165 mA/cm^−2^). When applied for the photoelectrocatalytic degradation of pharmaceuticals, the percentage removal of 80% and 86% were recorded for ciprofloxacin and sulfamethoxazole respectively. Overall, the reports from this research revealed that BiVO_4_/Ag_2_S electrode can be applied to oxidize recalcitrant pharmaceuticals in aqueous medium and pre-treated real pharmaceutical effluents and this can be achieved a reduced cost through the use of lower bias potential. In future works, enhancement of BiVO_4_/Ag_2_S performance for PEC water treatment will be studied using photoactive cathode.

## Supplementary information

Supplementary information.
