# Supplementary material for: Towards visible light driven photoelectrocatalysis for water treatment: Application of a FTO/BiVO4/Ag2S heterojunction anode for the removal of emerging pharmaceutical pollutants
Source: Sci Rep. 2020 Mar 24;10:5348. doi: 10.1038/s41598-020-62425-w (PMC7093548; doi:10.1038/s41598-020-62425-w)
Supplement: Supplementary file 1 — Supplementary information. [file 41598_2020_62425_MOESM1_ESM.docx]

**Towards visible light driven photoelectrocatalysis for water treatment: Application of a FTO/BiVO_4_/Ag_2_S heterojunction anode for the removal of emerging pharmaceutical pollutants**

Benjamin O. Orimolade^a^, Omotayo A. Arotiba^a,b,^*

*^a^Department of Chemical Sciences, University of Johannesburg, South Africa^[[1]](#footnote-1)^*

*^b^Centre for Nanomaterials Science Research, University of Johannesburg, South Africa*

**Corresponding author: oarotiba@uj.ac.za*

**Supplementary files**





**Figure S1: Kinetic plots for the degradation of ciprofloxacin**





**Figure S2: Kinetic plots for the degradation of sulfamethoxazole**

1. Formerly known as Department of Applied Chemistry, University of Johannesburg, South Africa [↑](#footnote-ref-1)
